# Nutrition and Its Impact on Quality of Life in Patients with Brain Tumors: A Scoping Review

**DOI:** 10.3390/cancers18030392

**Published:** 2026-01-27

**Authors:** Jude Banihani, Stephanie Cramer, Jethro Hu

**Affiliations:** Cedars-Sinai Medical Center, Los Angeles, CA 90048, USA; jude.banihani@cshs.org (J.B.); stephanie.cramer@cshs.org (S.C.)

**Keywords:** nutrition, brain cancer, quality of life, diet

## Abstract

**Background/Objective**: Nutrition plays a significant role in quality of life (QoL); however, information regarding the effects of nutritional interventions for brain tumors patients is limited. The objective of this scoping review is to analyze existing literature on nutrition and its impact on QoL in brain tumor patients. **Methods**: Using the PubMed database in July 2025, this scoping review identified original studies written in English using the keywords: nutrition, brain cancer, QoL, and diet. Using the ChatGPT5 prompts: “*I’m looking for journal articles and clinical trials with brain cancer, nutrition, quality of life*” in July 2025 and “*I’m looking for research articles assessing quality of life in brain tumor patients on ketogenic diet*” in December 2025, additional articles were identified. Studies investigating the implementation of nutritional interventions (specific diets, supplements) and effects on QoL in brain tumor patients of all ages were included. Reviews were not included, but original articles cited within the reviews were. Articles were reviewed by the authors for selection, and a total of twelve articles pertaining to eleven studies were identified. **Results**: Nine studies focused on the utilization of ketogenic diets (KD). One study pertained to protein supplementation, and another study discussed enteral and parental nutrition. Six studies reported positive effects on QoL, three reported no statistical significance, and one study reported a negative effect. One study had mixed results of a positive effect in one area and no statistical significance effects in another. Universal conclusions on QoL were limited since many studies had small sample sizes, lacked control subjects, and utilized self-reporting rather than standardized assessment tools. **Conclusions**: This review revealed that the evidence regarding the effect of KD on QoL in brain tumor patients is encouraging but limited. Data regarding the impact of other dietary interventions is insufficient to draw conclusions. Additional research is necessary. Future studies should consider (1) increasing recruitment to yield larger sample sizes, (2) standardizing nutritional interventions, (3) utilizing control subjects, and (4) utilizing standardized assessment tools.

## Simple Summary

Nutrition plays a key role in quality of life; however, there is limited existing literature regarding the benefits of specific nutritional interventions on the quality of life in patients with brain tumors. The aim of this scoping review is to provide a comprehensive analysis of the original studies exploring nutrition and its impact on the quality of life in patients with brain tumors. The existing literature on this topic is limited, and universal conclusions regarding quality of life are limited due to small sample sizes, the lack of control subjects, and the utilization of self-reporting rather than standardized assessment tools. We highlight areas in need of further exploration to help aid in designing future studies on this topic, in hopes of yielding results that may benefit or improve the quality of life in this patient population.

## 1. Introduction/Background

Primary brain and other central nervous system (CNS) tumors are a heterogeneous group of neoplasms that contribute substantially to cancer burden in the United States. Although often considered rare, malignant primary brain tumors are among the more prevalent cancers in individuals under 65 years of age. They represent the most common cancer among adolescents and young adults (AYAs) aged 15–39 years, the second most common cancer in children aged 0–14 years, and remain within the top five cancer types in adults aged 40–65 years [1].

Supportive care, including nutrition, is essential for maintaining quality of life and treatment tolerance in oncology. However, specific evidence regarding nutritional interventions for brain tumor patients remains limited [2]. Although brain tumor patients have a lower overall incidence of malnutrition compared to patients with other cancers—17.6% compared to 30% to 85%—the direct impact that brain tumors can have on cognitive, neurological, and physical function poses unique challenges [3]. For example, tumors that frequently affect the posterior fossa—diffuse midline brainstem glioma, medulloblastoma, pilocytic astrocytoma, etc., can cause dysphagia and nausea through direct effects on brainstem structures. Other, more general neurologic symptoms—e.g., headaches, fatigue, altered mentation—also can impact nutritional status. These effects are further compounded by treatment-related adverse effects, including fatigue, nausea, and loss of appetite [3,4].

Despite these challenges, many brain tumor patients express great interest in dietary interventions; in one survey, for example, over 70% of brain tumor patients expressed interest in implementing a modified ketogenic diet [5]. Indeed, in recent years, nutritional interventions (including dietary approaches) for brain tumor patients have been the subject of several studies. Most of these have been small and relatively limited in scope and generalizability, primarily consisting of case reports, retrospective case series, or small clinical trials. Several of these studies focus on the ketogenic diet as a potential metabolic therapy, aiming to alter tumor cell metabolism [6,7]. While some of these studies suggest feasibility, potentially therapeutic metabolic effects, and possible improvements in quality of life, formal assessment of quality of life in these studies has been limited [8,9].

Given the small sample sizes, methodological heterogeneity, and narrow focus of existing research, there remains a need to review the current evidence regarding nutrition and quality of life in patients with brain tumors. This scoping review aims to (1) map the range and characteristics of studies addressing nutrition and quality of life in this population; (2) identify key nutritional assessments and interventions employed; and (3) highlight evidence gaps and future research directions. By integrating findings across nutrition, oncology, and supportive care, this review seeks to clarify the current landscape and provide an overview of how nutrition may improve quality of life by influencing both physical and psychosocial dimensions of well-being in neuro-oncology.

## 2. Methods

**Figure 1:**
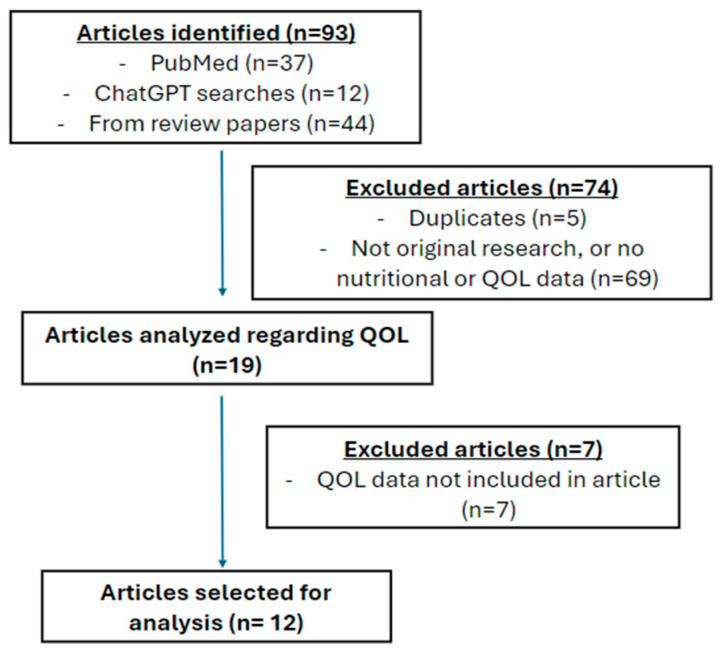
PRISMA flowchart. Article selection process. Twelve articles pertaining to eleven studies were selected for this scoping review.

A scoping review was conducted using PubMed beginning in July 2025 using the keywords: nutrition, brain cancer, quality of life, and diet. This search yielded thirty-seven articles, four of which met the inclusion criteria. Additional articles on this topic were identified using the following ChatGPT5 prompts: “*I’m looking for journal articles and clinical trials with Brain Cancer, nutrition, and quality of life*” (last assessed in July 2025) and “*I’m looking for research articles assessing quality of life in brain tumor patients on ketogenic diet*” (last assessed in December 2025). Studies investigating the implementation of any nutritional interventions and their effect on quality of life in brain tumor patients were used. There was no exclusion criterion for nutritional interventions included, and our search included various interventions such as specific diets, supplementation use, etc. Pediatric and adult populations were included. Only studies written in English and with original research (clinical trials, case reports, retrospective studies) were analyzed. Although review articles were not included, articles cited from these papers pertaining to our topic of interest were. Nineteen articles were initially identified by authors independently. Articles were then analyzed by two authors for final selection. Two articles pertaining to the same study were included [10,11]. A total of twelve articles investigating eleven studies were selected for this scoping review (Figure 1 PRISMA flowchart). Details pertaining to the selected articles can be found in Table 1.This scoping review followed the Preferred Reporting Items for Systematic Review and Meta-analyses (PRISMA) checklist and was not registered.

Additionally, to investigate ongoing and planned future studies on this topic, our team searched for recruiting and yet to be recruiting clinical trials investigating the effects of diet interventions on the quality of life in patients with brain tumors. Using the search engine on the clinicaltrials.gov website, the term “brain tumors” was used under the “condition/disease” section. Searches under the “intervention/treatment” section were used with the following terms: diet, nutrition, diet interventions, ketogenic diet, supplements and/or medical foods, fasting mimicking diet, and fasting. A total of forty-five active clinical studies were yielded. After removal of duplicate studies and studies not pertaining to quality of life, patients with brain tumors, and/or investigating diet intervention, there were four clinical trials that we have included in this scoping review. Table 2, shown below, provides a summary of these clinical trials, including the trial number, trial name, trial status, intervention, primary objective, site(s) of the clinical trial, the quality of life (QoL) measurement tool used, and the timing of the quality of life (QoL) assessment.

## 3. Results

Twelve articles analyzing eleven studies were selected. A summarized table of the characteristics of the selected articles, including study type, number of patients who started and completed the intervention, intervention studied, primary objectives, assessment tools utilized, and quality of life (QoL) results, can be found in Table 1. Of these eleven studies, nine pertained to the utilization of ketogenic diets, which greatly varied among studies regarding the type and degree of caloric restriction that was utilized. One study pertained to protein supplementation use, and another study discussed the difference between oral nutrition counseling and enteral and parenteral nutrition. Eight studies pertained to adult patient populations with brain tumors, while three articles pertained to pediatric populations. Within these patient populations, various types of brain tumors were studied. Five studies focused on patients with glioblastoma multiforme (GBM). One study evaluated patients with medulloblastoma and supratentorial primitive neuroectodermal tumors, while another study focused on malignant astrocytoma tumors. Three studies included patients with a mixture of different brain tumor types. Study design also varied among the selected articles and consisted of three retrospective studies, eight clinical trials, and a case series. The duration of the interventions greatly varied, ranging from 9 days to up to 12 months, while the duration of retrospective periods ranged from 3 months to up to a 10 year-period. The primary objectives investigated in these studies included feasibility, safety, tolerability, overall survival, retention rate at 3 months, percent change in body weight, progression-free survival, muscle strengthening and functional gain, and tumor glucose metabolism assessed by PET uptake at the tumor site. In addition to investigating these primary objectives, these articles also reported the effects of their studied nutritional intervention on quality of life. Five studies utilized descriptions to assess quality of life, while five studies utilized assessment tools. One article used both a case description and an assessment tool. The assessment tools utilized include the European Organization for Research and Treatment of Cancer (EORTC) QLQ-C30 questionnaire, EORTC QLQ BN-20 questionnaire, Beck Depression Inventory (BDI), Brief Fatigue Inventory (BFI), and Edmonton Symptom Assessment System (ESAS) assessment. Six studies reported positive effects on quality of life. Three studies reported no statistical significance. One study reported a negative effect on quality of life. The study utilizing protein supplementation had mixed results, indicating a positive effect on the physical metrics of pinch grasp and a 6 min walk test, but no statistical significance in the QoL-related BDI and BFI scores before and after the intervention.

## 4. Discussion

Diet is well-recognized as a potential mediator of oncogenesis. The World Health Organization estimates that thirty to fifty percent of cancers are related to modifiable lifestyle and environmental risk factors such as tobacco use, alcohol use, and diet [19]. Consumption of red meat and processed meat results in elevated exposure to carcinogenic nitrites, heterocyclic amines, and polycyclic aromatic hydrocarbons; dairy consumption results in higher blood IGF-1 levels; and obesity causes hyperinsulinemia and increased inflammation [19]. On the positive side, vegetables and fruits contain a plethora of phytochemicals that may have antineoplastic effects, including carotenoids, flavonoids, and phenols; high fiber intake reduces inflammation, promotes gut health, and potentially binds carcinogens [19]. Cancers related to the alimentary tract—colorectal, gastric, pancreatic, esophageal, oropharyngeal—have a particularly strong link to diet, but other cancers (including breast, uterine, and thyroid cancers) have an association as well [20]. Regarding brain tumors, a 2015 meta-analysis showed an association between obesity and meningioma, as well as increased risk of glioma in obese females but not obese males [21].

Despite the absence of a strong link between diet and the pathogenesis of brain tumors, many brain tumor patients are keenly interested in dietary and nutritional interventions as a possible means of improving therapeutic efficacy, functional outcomes, and quality of life. In this scoping review, we investigated existing literature on nutrition and its effect on brain tumor patients; however, the existing literature was limited. A large majority of the available studies evaluated the ketogenic diet, mostly focusing on its safety, feasibility, and metabolic effects. Although the ketogenic diet studies highlighted the feasibility and safety of the diet with minimal reported side effects, there is a relative paucity of data regarding the effects on quality of life for this patient population. The data regarding the effects of nutritional interventions on quality of life for patients with other cancer types is also limited, though slightly more robust. In Schmidt et al. 2011, sixteen patients with advanced metastatic tumors followed a ketogenic diet [22]. Patients in this study had ovarian, breast, parotid, osteosarcoma, esophageal, pancreatic, thyroid, colon, endometrial, lung, and stomach cancers [22]. Using the EORTCQLQ-C30 questionnaire, quality of life data from seven patients who followed dietary intervention for at least 2 months revealed stable global health status scores, slightly increased emotional functioning, and improved insomnia [22]. In another study, patients with locally advanced or metastatic breast cancer on a ketogenic diet were evaluated using the EORTC QLQ-C30 and IORTC QLQ-BR23 questionnaires [23]. In the ketogenic diet group, quality of life scores were higher than those of the control group at 6 weeks; however, there was no difference at 12 weeks [23].

In this scoping review, we identified eleven studies with at least some data regarding the quality-of-life effects of nutritional intervention for patients with brain tumors. Six studies reported positive effects on quality of life in areas such as mood and daily activities: of these, four utilized descriptive analysis only. Three studies reported no statistical significance, and one study reported a negative effect on quality of life, resulting in 3 of 20 participants discontinuing the diet. One study utilizing protein supplementation showed mixed results with evidence of a positive effect on the ability to perform pinch grasp and a 6 min walk, but no statistically significant effect on the BDI and BFI scores. Overall, approximately 50% of identified studies relied on descriptive quality of life assessment rather than formal assessment tools, limiting the validity of these results.

Despite the limitations in available data regarding the effect of nutritional interventions on quality of life for brain tumor patients at this time, it is still well-established that nutrition assessment is essential for identifying at-risk patients and initiating timely interventions. Evidence-based practices include the use of validated screening tools alongside comprehensive assessments that include anthropometrics, inflammation screening, and body composition analysis, as outlined by the American Society for Parenteral and Enteral Nutrition (ASPEN) systematic review on malnutrition screening and the European Society for Clinical Nutrition and Metabolism (ESPEN) Practical Guidelines on Clinical Nutrition in Cancer [24,25].

Nutritional assessment and interventions are also an important component of a broader holistic and integrative model of care that encompasses nutrition, physical activity, and complementary modalities to address both physical and psychosocial dimensions of well-being. The American Society of Clinical Oncology’s *Integrative Therapies in Cancer Care* update highlights the growing role of diet, mind–body practices, and lifestyle interventions in symptom management and quality of life enhancement [26]. Similarly, evidence suggests that physical activity and nutrition interventions may synergistically improve outcomes in older adults with cancer, while dietary modifications may enhance treatment efficacy and mitigate inflammation [27,28]. Complementary approaches have also been explored for cancer-related fatigue, a leading quality of life concern in brain tumor patients [26].

This scoping review also had its limitations. Our literature search utilized only one traditional database in addition to the use of ChatGPT. Many of the available studies assessed nutritional interventions over a small time period; future research should consider evaluating nutritional interventions for a longer duration of time and incorporate quality of life monitoring beyond the duration of the intervention to assess the long-term effects of these nutritional interventions. Although some articles used assessment tools such as the EORTC QLQ C30 and BN20 questionnaires, many articles depended on self-reporting for their analysis. Although the information produced was valuable, future research should incorporate standardized assessment tools that are validated in assessing quality of life. This approach would enable comparison not only between intervention versus control arms within a study, but also across different studies.

At this time, due to limited data, lack of use of standardized assessment tools, and small sample sizes of the available studies, universal conclusions cannot be made regarding the effects of nutritional interventions on the quality of life of brain tumor patients. Studies investigating the effects of a particular dietary approach, such as the ketogenic diet, are also limited by heterogeneity of diet implementation both within and across studies [14]. Future studies investigating the effect of nutritional interventions on quality of life in brain tumor patients should focus on the use of standardized and valid assessment tools rather than description. Future studies should also create studies with larger sample sizes and, when possible, incorporate study designs that utilize control groups to facilitate comparison.

Looking into ongoing and future studies, our search on the ClinicalTrials.gov website yielded four active clinical trials with a component of their study design assessing the effects of a nutritional intervention on the quality of life in patients with brain tumors (Table 2). All these clinical trials will be investigating the effects of the ketogenic diet and are currently recruiting participants. Two of the four clinical trials will be utilizing standardized assessment tools, such as the Functional Assessment of Cancer Therapy—Brain, Acromegaly Quality of Life questionnaire, Patient-Assessed Acromegaly Symptom Questionnaire, and D-KETOcheck questionnaires. One clinical trial will be utilizing a non-validated questionnaire consisting of open and closed questions to be administered to the parents of their pediatric patients. Another clinical trial will be utilizing a combination approach with standardized assessment tools—namely the Heart Hope Index, SF-36, cancer-specific FACT surveys, Brief Pain Inventory, and Brief Fatigue Inventory questionnaires in addition to self-report diaries. Through the use of these standardized assessment tools, we hope these future clinical trials will yield additional results on the topic of quality of life and nutritional interventions in brain tumor patients and provide further insight and recommendations on how to further support the care of this specific patient population.

## 5. Conclusions

This scoping review revealed encouraging yet limited information regarding the effects of nutritional interventions on quality of life for brain tumor patients. Most of the identified studies utilized the ketogenic diet as the nutritional intervention. Universal conclusions of these interventions were limited, as the studies relied on self-reporting rather than the use of standardized assessment tools, had small sample sizes, and lacked control subjects. Due to the limited existing data, additional research on this topic is needed to answer key questions regarding the implementation of nutritional interventions for this patient population. Future studies should focus on (1) utilizing standardized assessment tools, (2) increasing recruitment to yield larger sample sizes, and (3) utilizing control subjects for easier comparisons.

## Figures and Tables

**Table 1:** Summarized table of the characteristics of the selected articles, including study type, number of patients who started and completed the intervention, intervention studied, primary objectives, assessment tools utilized, and QOL results. Results indicated with “↑” arrow demonstrated increase effects, while results indicated with “↓” arrow demonstrated decreased effects.

Article Citation	Type of Study	Patient Population	# Started Intervention	# Completed the Intervention	Intervention	Primary Objective	Duration of Study	Assessment Tools Used	QOL Results
Bakish J et al. 2003 [12]	Retrospective case series	Children with newly diagnosed medulloblastoma and supratentorial primitive neuroectodermal tumor	103	103	Oral diet counseling vs. enteral nutrition vs. parenteral nutrition	Percent change in body weight	Over 10 years	Descriptive	Enteral nutrition facilitates weight gain and therefore improves the quality of life for the patient and the caregiver
Rieger J et al. 2014 [6]	Clinical trial	Adults with recurrent GBM	20	17	KD with no caloric restriction	Feasibility	3–13 weeks (until disease progression)	Descriptive	3 patients discontinued due to the diet negatively affecting QOL
Van der Louw et al. 2019 [13]	Clinical trial	Adult patients with GBM	9	6	KD (4:1 ratio)	Feasibility	14 weeks	EORTC QLQ-C-30 questionnaire	No significant change over the course of the study
Klein P et al. 2020 [14]	Clinical trial	Adult patients with either newly diagnosed GBM treatment or recurrent GBM	8	5	KD (4:1 ratio)	Feasibility, safety, and tolerability	6 months	Descriptive	Case description reporting “excellent quality of life”
Martin-McGill KJ et al. 2020 [5]	Clinical trial with embedded qualitative study	Adult patients with recently diagnosed GBM	10	4 completed 3 months, 3 completed 12 months	Modified KD or medium-chain triglyceride KD	Retention rate at three months	3 months (primary end point) with an option to continue for a total of 12 months (secondary end point)	EORTC QLQ C30 and BN20 questionnaires	Patients who maintained KD had a Global Health Status above the brain cancer reference value, while patients who withdrew fell below the reference value
Nebeling LC et al. 2020 [15]	Case reports	Pediatric patients with advanced stage malignant astrocytoma tumors	2	2	MCT oil-based KD	Tumor glucose metabolism assessed by PET uptake at the tumor site	8 weeks	Descriptive	One patient with “marked progress in skill development (gait, mobility, speech, hand coordination) and mood” “QOL greatly improved”
Panhans CM et al. 2020 [16]	Retrospective case series	Adult patients with GBM, astrocytoma, or oligodendroglioma	12	8	KD (3:1 ratio)	Feasibility and safety	120 days	ESAS assessment, descriptive	Reported improvement in fatigue in 8/12 patients and in mood in 7/12 patients. Patient case descriptions of improved QOL
Voss M, Wagner M et al. 2020, 2022 [10,11]	Clinical trial	Adult patients with recurrent malignant gliomas (GBM, gliosarcoma, or malignant progression of lower grade glioma)	23	20	Calorically restricted KD and intermittent fasting	Progression-free survival	9 days	EORTC QLQ C30 and BN20 questionnaires	No significant difference between the two groups during treatment or at later follow-up
Perez, A. et al. 2021 [17]	Retrospective case series	Children with diffuse intrinsic pontine glioma	6	5	KD (varied)	Feasibility, safety, and overall survival	3 months	Descriptive	“Some cases with improvements of some symptoms resulting in a better participation in daily life activities”
Cho KH et al. 2024 [18]	Clinical trial	Adult with different types of brain tumors	30	20	Protein supplements	Muscle strengthening and functional gain	6 weeks	Beck Depression Inventory (BDI) and Brief Fatigue Inventory (BFI)	No statistical significance in BDI and BFI before and after assessment. Improved pinch grasp and 6 min walk in the protein supplementation group
Amaral LJ et al. 2025 [8]	Clinical trial	Adult patients with recently diagnosed GBM	20	17	KD (3:1 ratio)	Safety	16 weeks	EORTC QLQ-C30 and QLQ BN-20 questionnaires	No statistically significant changes over the study. Non-significant results: Appetite ↓ Constipation ↑ N/v ↑ Overall QoL ↑ Seizure severity ↓ Headaches ↓

**Table 2:** Active clinical trials on the effects of diet interventions on quality of life in patients with brain tumors as found on the ClinicalTrials.gov website. This table provides a summary of these trials, including trial number, trial name, trial status, intervention, primary objective, site(s) of the clinical trial, QoL measurement tool used, and the timing of QoL assessment.

Trials Number	Trial Name	Trial Status	Intervention	Primary Objective	Site(s)	QoL Measurement	Timing of QoL Assessment
NCT05428852	Keto-Brain: Investigating the Use of Ketogenic Diets in Brain Metastases	Recruiting	Ketogenic diet	Feasibility	Columbus, Ohio, United States, 43210	Hearth Hope Index (HHI), SF-36, cancer-specific FACT surveys, Brief Pain Inventory, Brief Fatigue Inventory, and self-report diaries	Baseline up to 16 weeks
NCT05564949	A Ketogenic Diet as a Complementary Treatment for Patients with High-grade Gliomas and Brain Metastases	Recruiting	Ketogenic diet	Overall survival	Athens, Greece, 12462. Chaïdári, Athens, Greece, 12461	Functional Assessment of Cancer Therapy—Brain (FACT-Br)	Baseline, 3 months, 6 months, 12 months
NCT06309251	Effectiveness and Impact on the Quality of Life of the Ketogenic Diet in Pediatric Patients	Recruiting	Ketogenic diet	Nutritional adequacy	Milan, MI, Italy, 20154. Pavia, PV, Italy, 27100. Roma, RM, Italy, 00165	Non-validated questionnaire administered to parents with qualitative open and closed questions	6 months (12 months optional)
NCT06949891	KETOgenic Diet Therapy in Patients with ACROmegaly	Recruiting	Ketogenic diet	Difference in IGF-1	Rotterdam, Netherlands, 3015 GE	Acromegaly Quality of Life questionnaire. Patient-Assessed Acromegaly Symptom Questionnaire. D-KETOcheck questionnaire	At baseline, 3 months, and 6 months.

## Data Availability

The original contributions presented in this study are included in the article/Supplementary Materials. Further inquiries can be directed to the corresponding author.

## Supplementary Materials

The following supporting information can be downloaded at: https://www.mdpi.com/article/10.3390/cancers18030392/s1.

## Abbreviations

QoL	Quality of Life
KD	Ketogenic diet(s)
CNS	Central nervous system
AYA	Adolescents and young adults
PRISMA	Preferred Reporting Items for Systematic Review and Meta-analyses
ASPEN	American Society for Parenteral and Enteral Nutrition
ESPEN	European Society for Clinical Nutrition and Metabolism
GBM	Glioblastoma multiforme
PET	Positron Emission Tomography
EORTC	European Organization for Research and Treatment of Cancer
BDI	Beck Depression Inventory
BFI	Brief Fatigue Inventory
ESAS	Edmonton Symptom Assessment System
MCT	Medium Chain Triglycerides
